# Supplementary material for: Risk Factors for Exercise-Associated Sudden Cardiac Death in Thoroughbred Racehorses
Source: Animals (Basel). 2022 May 18;12(10):1297. doi: 10.3390/ani12101297 (PMC9137751; doi:10.3390/ani12101297)
Supplement: Supplementary file 1 [file animals-12-01297-s001.zip › animals-1723783-supplementary.pdf]

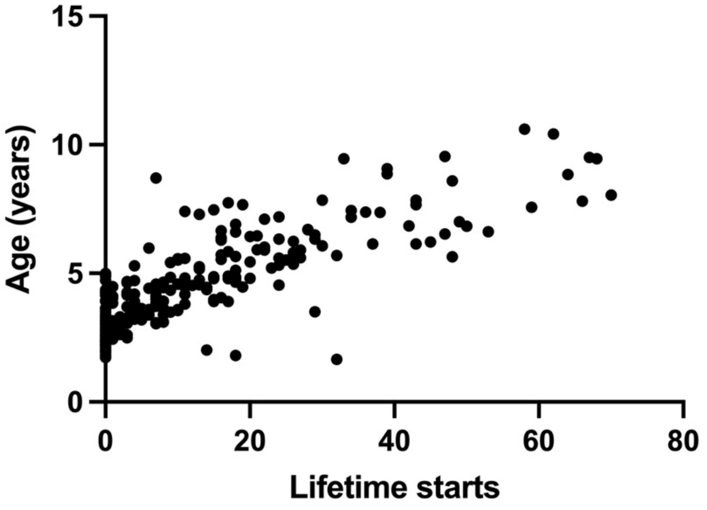

Figure S1. Correlation between Age and lifetime starts. Spearman *r* 0.81, 95%CI 0.76–0.85, $p < 0.0001$.

Table S1. Pulmonary lesion grade at post-mortem for 34/57 (59.6%) examined SCD horses and 129/188 (67%) examined OFI horses.

Pulmonary lesion	SCD *n* = 34	OFI *n* = 129
**Pulmonary oedema**		
Grade 0	2 (6%)	22 (17%)
Grade 1	6 (18%)	34 (26%)
Grade 2	3 (9%)	30 (23%)
Grade 3	6 (18%)	25 (19%)
Grade 4	18 (53%)	17 (13%)
**Pulmonary congestion**		
Grade 0	2 (6%)	19 (15%)
Grade 1	3 (9%)	28 (22%)
Grade 2	10 (29%)	35 (27%)
Grade 3	14 (41%)	34 (26%)
Grade 4	6 (18%)	13 (10%)
**Acute Pulmonary Haemorrhage**		
Grade 0	4 (12%)	59 (46%)
Grade 1	7 (21%)	42 (33%)
Grade 2	12 (35%)	21 (16%)
Grade 3	6 (18%)	3 (2%)
Grade 4	6 (18%)	4 (3%)
**Chronic Pulmonary Haemorrhage**		
Grade 0	24 (71%)	97 (75%)
Grade 1	9 (26%)	22 (17%)
Grade 2	1 (3%)	6 (5%)
Grade 3	0 (0%)	2 (2%)
Grade 4	1 (3%)	2 (2%)

Table S2. Multiple logistic regression of relationship between each included variable and SCD with age retained.

Variable Continuous, median (IQR)	SCD *n* = 57	OFI *n* = 188	***β*** coeff	Log odds ratio	OR 95% CI	*p* value

Categorical, proportion (%[95% CI])						
Sex						
Gelding (REF)	27 (47[35–60]%)	94 (50[43–57]%)				
Female	21 (37[25–50]%)	48 (25[10–32]%)	–0.03	0.97	0.45–2.05	0.93
Entire male	9 (16[8–28]%)	46 (25[19–31]%)	0.96	0.39	0.15–0.96	0.05
Sire hemisphere of origin						
Southern hemi (REF)	35 (65[51–76]%)	97 (52[45–69]%)				
Northern hemi	19 (35[24–49]%)	89 (48[41–55]%)	–0.31	0.73	0.37–1.40	0.35
Exercise type						
Racing (REF)	15 (26[17–39]%)	106 (56[49–63]%)				
Training	42 (74[63–81]%)	82 (44[37–51]%)	0.96	2.61	1.25–5.66	0.01
Lifetime starts	3 (0–9.0)	9 (0–22.8)	–0.05	0.95	0.90–0.99	0.04
Age	3.58 (3.05–4.36)	4.5 (3.12–6.02)	0.09	1.10	0.78–1.55	0.59

Tjur's $r^2 = 0.11$, area under ROC curve (95%CI) = 0.73 (0.66–0.80), $p < 0.0001$. Hosmer-Lemeshow statistic 12.75, $p = 0.12$.

Table S3. Multiple logistic regression of relationship between each included variable and SCD with lifetime starts discarded.

**Variable Continuous, median (IQR) Categorical, proportion (%[95% CI])**	**SCD** *n* = 57	**OFI** *n*= 188	***β* coeff**	**Log odds ratio**	**OR 95% CI**	***p* value**
Sex						
Gelding (REF)	27 (47[35–60]%)	94 (50[43–57]%)				
Female	21 (37[25–50]%)	48 (25[10–32]%)	0.15	1.16	0.56–1.46	0.68
Entire male	9 (16[8–28]%)	46 (25[19–31]%)	–0.91	0.40	0.15-0.99	0.06
Sire hemisphere of origin						
Southern hemi (REF)	35 (65[51–76]%)	97 (52[45–69]%)				
Northern hemi	19 (35[24–49]%)	89 (48[41–55]%)	–0.27	0.76	0.39–1.46	0.42
Exercise type						
Racing (REF)	15 (26[17–39]%)	106 (56[49–63]%)				
Training	42 (74[63–81]%)	82 (44[37–51]%)	1.12	3.05	1.50–6.45	<0.01
Age	3.58 (3.05–4.36)	4.5 (3.12–6.02)	–0.18	0.84	0.66–1.04	0.12

Tjur's $r^2 = 0.10$, area under ROC curve (95%CI) = 0.73 (0.66–0.80), $p < 0.0001$. Hosmer-Lemeshow statistic 11.98, $p = 0.15$.
